# SNHG17 promotes the proliferation and migration of colorectal adenocarcinoma cells by modulating CXCL12-mediated angiogenesis

**DOI:** 10.1186/s12935-020-01621-0

**Published:** 2020-11-26

**Authors:** Yang Liu, Qinshan Li, Dongxin Tang, Mengxing Li, Peng Zhao, Wenxiu Yang, Liping Shu, Jishi Wang, Zhixu He, Yanju Li, Feiqing Wang

**Affiliations:** 1 Department of Science and Education, The First Affiliated Hospital of Guizhou University of Traditional Chinese Medicine, No. 71 Bao Shan North Road, Yunyan District, Guiyang, 550001 Guizhou China; 2 Department of Hematology, Affiliated Hospital of Guizhou Medical University, No. 28 Guiyi Street, Yunyan District, Guiyang, 550004 Guizhou China; 3 National & Guizhou Joint Engineering Laboratory for Cell Engineering and Biomedicine Technique, Guizhou Medical University, Guiyang, 550004 Guizhou China; 4 Department of Pediatrics, Affiliated Hospital of Zunyi Medical University, Zunyi, 563006 Guizhou China

**Keywords:** SNHG17, miR-23a-3p, CXCL12, Angiogenesis, Colorectal adenocarcinoma

## Abstract

**Background:**

Colorectal adenocarcinoma (CRA) is one of the leading causes of cancer-related deaths in the world. Long non-coding RNAs (lncRNAs) have been implicated to be effective regulators in the disease course of human cancers, including CRA. Small nucleolar RNA host gene 17 (SNHG17) belongs to lncRNAs, and it has been reported in breast cancer and gastric cancer. However, the function of SNHG17 and its mechanism in CRA progression remain largely unknown. In this study, we attended to shedding some light on the role of SNHG17 in CRA.

**Methods:**

RT-qPCR was used to assess SNHG17 expression in CRA cells. CCK-8 assay, colony formation and transwell assay were carried out to detect the regulatory effect of SNHG17 silencing on CRA cell proliferation and migration. The angiogenesis of SNHG7-downregulated CRA cells was analyzed by tube formation assay. Mechanism experiments were conducted to identify the interaction between miR-23a-3p and SNHG17 or C-X-C motif chemokine ligand 12 (CXCL12).

**Results:**

SNHG17 possessed with high expression in CRA cells. Knockdown of SNHG17 caused the inhibition on CRA cell proliferation and migration. SNHG17 promoted CRA cell proliferation and migration by sponging miR-23a-3p to upregulate CXCL12.

**Conclusion:**

SNHG17 promotes the proliferation and migration of CRA cells by inhibiting miR-23a-3p to modulate CXCL12-mediated angiogenesis.

## Background

Colon cancer (CC) can be pathologically divided into colorectal adenocarcinoma (CRA), mucinous adenocarcinoma and undifferentiated cancer, among which CRA accounts for the majority of all CC cases [1–3]. CRA refers to a malignant tumor originally occurring in colon epithelial cells, which possesses with significantly high mortality each year [4]. There is a report reflected that CRA mainly occurs in the group of older people [5]. Some adjuvant therapeutic methods such as chemotherapy and radiotherapy are widely used to decrease the mortality of CRA patients, but the survival rate still remains unsatisfactory [6, 7], Hence, exploring novel target is necessary and essential for prolonging the life of patients with CRA.

Long noncoding RNAs (lncRNAs) are acknowledged to involve in various physiological processes through chromatin remodeling, histone modification, and DNA methylation even as transcription factors or enhancers [8–10]. LncRNAs can affect the progression of human cancers, including CRA [11]. In this regard, most of lncRNAs are considered as the potential therapeutic targets for tumors [12]. LncRNAs have also been reported to be oncogenic or tumor-suppressing in CRA. For instance, lncRNA MEG3 has been proven to exert as a tumor-suppressing gene to depress CRA cell proliferation [13]. LINC01638 is up-regulated in CRA cells and promotes cell proliferation [14]. Small nucleolar RNA host gene 17 (SNHG17) belongs to lncRNA family, which has been elucidated in human cancers due to its oncogenic potential. For example, SNHG17 accelerates the progression of gastric cancer by downregulating p15 and p57 [15]. Recently, SNHG17 was reported as an oncogene in prostate cancer [16], breast cancer [17], glioma [18]. However, the mechanism by which SNHG17 exerts functions in CRA remain to be unmasked.

In current study, we aimed to explore the function of SNHG17 in CRA and unmask its underlying mechanism as well.

## Methods

### Cell lines and culture

Human colon epithelial cell line FHC (ATCC; Manassas, VA, USA) was routinely grown in DMEM: F12 Medium (ATCC). Human umbilical vein endothelial cells (HUVECs; ATCC) were maintained in F-12K Medium (ATCC). Besides, the colorectal cancer cell line SW480 (ATCC) was cultured in ATCC-formulated Leibovitz’s L-15 Medium. LoVo (ATCC) was cultured in ATCC-formulated F-12 K Medium. RKO (ATCC) was cultured in ATCC-formulated Eagle’s Minimum Essential Medium. HCT116 (ATCC) was cultured in ATCC-formulated McCoy’s 5a Medium Modified. Fetal bovine serum (FBS, 10%) was procured from Gibco (Rockville, MD, USA) and used for cell culture in a humidified atmosphere containing 5% CO_2_ at 37 °C. Cells were passaged when the confluence was more than 80%.

### Real-time quantitative polymerase chain reaction (RT-qPCR)

Total RNA was isolated initially by use of TRIzol Reagent (Invitrogen, Carlsbad CA, USA), and then converted into cDNA in the presence of PrimeScript Reverse Transcriptase Kit (Takara, Shiga, Japan). ABI 7500 Real-Time PCR system (Applied Biosystems, Foster City, CA, USA) was used for each PCR reaction. Next, RT-qPCR experiment was completed by use of SYBR Green PCR Kit (Takara). All gene expressions were processed based on the comparative change-in-cycle method (ΔΔCt), with GAPDH or U6 as internal control. Sequences for all primers were provided in Additional file 1: Table S1. Each experimental procedure was repeated at least three times.

### Cell transfection

To silence SNHG17 expression, the specific shRNAs (sh/SNHG17#1/2/3) and the negative control (NC) were constructed by GenePharma (Shanghai, China). To overexpress expressions of SNHG17 and CXCL12, the full length of SNHG17 and CXCL12 cDNA sequences were separately inserted into pcDNA3.1 vectors (Invitrogen). The empty vectors were utilized as NC. The miR-23a-3p mimics and miR-23a-3p inhibitor were synthesized by Ribobio (Guangzhou, China), as well as NC mimics and NC inhibitor. RKO and HCT116 cells in 6-well plates (1 × 10^6^ cells/well) were prepared for 48 h of transfection using Lipofectamine 3000 (Invitrogen). Sequences used in transfection were shown in Additional file 1: Table S1. Each experimental procedure was repeated at least three times.

### Cell counting Kit-8 (CCK-8) assay

Transfected cells were incubated until the confluence reached to 80%-90%, 5 × 10^3^ RKO and HCT116 cells were seeded into the each well of 96-well plates. CCK-8 solution (10 μL; Dojindo, Kumamoto, Japan) was added into each well at several time points (24 h, 48 h, 72 h) and incubated for 2 h at 37 °C in 5% CO_2_. Cell viability was examined via observing the optical density value at 450 nm using spectrophotometer (Thermo Fisher Scientific, Waltham, MA, USA). The assay was carried out for three times.

### Colony formation assay

After required transfections, 500 RKO and HCT116 cells were prepared in the each well of 6-well plates and incubated for 14 days. After that, 4% paraformaldehyde (PFA) was added to fix cells for 30 min, 0.5% crystal violet solution was added to stain cells for 5 min. The resulting colonies were counted manually. The experiment was conducted in triplicate.

### Transwell migration assay

transfected RKO and HCT116 cells were collected and placed into the upper chamber of 24-well Transwell inserts (Corning Incorporated, Corning, NY, USA) at 2 × 10^4^ cells per well. Lower chamber was added with the complete culture medium containing 10% FBS. After cells were incubated for 24 h, the migrated cells were fixed with PFA for 30 min, and then cells were treated with 0.5% crystal violet for visualizing. Five random fields were observed using optical microscope (Olympus, Tokyo, Japan; magnification, ×200). The assay was performed for three times.

### Wound healing assay

RKO and HCT116 cells, 1 × 10^6^ cells were collected after transfection, and then cultured until about 90% cell confluence. The wounds were made by 200-μL pipette tip. At 0 h and 24 h of cell culture in serum-free medium, the wound closure was separately assessed under microscope. Each experimental procedure was repeated at least three times.

### Subcellular fractionation

Subcellular fractionation assay was undertaken in RKO and HCT116 cells in line with the protocol of the nuclear or cytoplasmic Isolation Kit (Biovision, San Francisco, CA, USA). Cell fractionation buffer and cell disruption buffer were used for isolating cell cytoplasm and cell nucleus. After centrifugation, the content of SNHG17 in two fractions was detected by RT-qPCR, with GAPDH and U6 as internal references. The assay was carried out three times.

### FISH

Ribo™ Fluorescent in Situ Hybridization Kit (Ribobio) was applied to conduct FISH assay. After fixation with 4% PFA for 30 min, RKO and HCT116 cells were treated with 0.5% Triton X-100 for 5 min at 4 °C. Then, cells were incubated with the specific FISH probe to SNHG17 (Ribobio) in hybridization buffer. The sequences for SNHG17 probe were listed in Additional file 1: Table S1. Thereafter, DAPI solution was added for nuclear staining. The fluorescent analysis was conducted using a fluorescence microscope (Olympus). This assay was executed in triplicate.

### RNA pull down assay

At first, the complementary probe for SNHG17 and negative control probe was labeled with biotin and synthesized. Pierce Magnetic RNA–Protein Pull-Down Kit was acquired to conduct RNA pull down assay, following the user guide (Thermo Fisher Scientific). RKO and HCT116 cells were lysed in RIPA lysis buffer, and then the biotinylated probes for SNHG17 or miR-23a-3p were cultured with cell extracts. The cell lysates were incubated with proteinase K to remove proteins and terminate the crosslinking. After RNAs were extracted with Trizol reagent, the RNA enrichment was detected by RT-qPCR. This assay was executed in triplicate.

### RNA immunoprecipitation (RIP)

RIP assay was undertaken for RNA interaction, following the established protocol of Magna RIP™ RNA-Binding Protein Immunoprecipitation Kit (Millipore, Bedford, MA, USA). Cell lysates were incubated with the anti-Ago2 (1: 200; Millipore) or control anti-IgG (1: 200; Millipore) on 30 μl of magnetic beads at 4 °C overnight. After that, the recovered immunoprecipitates were analyzed using RT-qPCR. This assay was executed in triplicate.

### Luciferase reporter assay

The fragments of SNHG17 and CXCL12 3′UTR covering the wild-type or mutant miR-23a-3p interacting sites were inserted into pmirGLO dual-luciferase vectors (Promega, Madison, WI, USA). RKO and HCT116 cells were co-transfected with the constructed pmirGLO vectors and indicated transfection plasmids for 48 h using Lipofectamine 3000. Afterwards, Dual-luciferase reporter assay system (Promega) was utilized to detect the luciferase activity. Results were normalized to Renilla luciferase activity. This assay was executed in triplicate.

### Tube formation assay

HUVECs (2.5 × 10^4^ cells) were cultured in the presence of the conditioned medium for RKO and HCT116 cells (RKO-CM and HCT116-CM) in 96-well plates coated with Matrigel membrane (BD Biosciences, Franklin Lakes, NJ, USA) for 6 h at 37 °C. Tube formation was visualized under light microscope (Olympus). Number of branches was counted for analysis. This assay was carried out in triplicate.

### Statistical analysis

Each procedure in all experiments was conducted in triplicate. The data were displayed as the mean ± SD. Group difference in each assay was estimated with one-way analysis of variance (ANOVA; more than two groups) or Student’s *t* test (two groups). Statistical analysis was accomplished with GraphPad PRISM 6 (GraphPad, San Diego, CA, USA). Data were considered statistically significant when p < 0.05.

## Results

### SNHG17 strengthens the viability, proliferation and migration of CRA cells

**Fig. 1:**
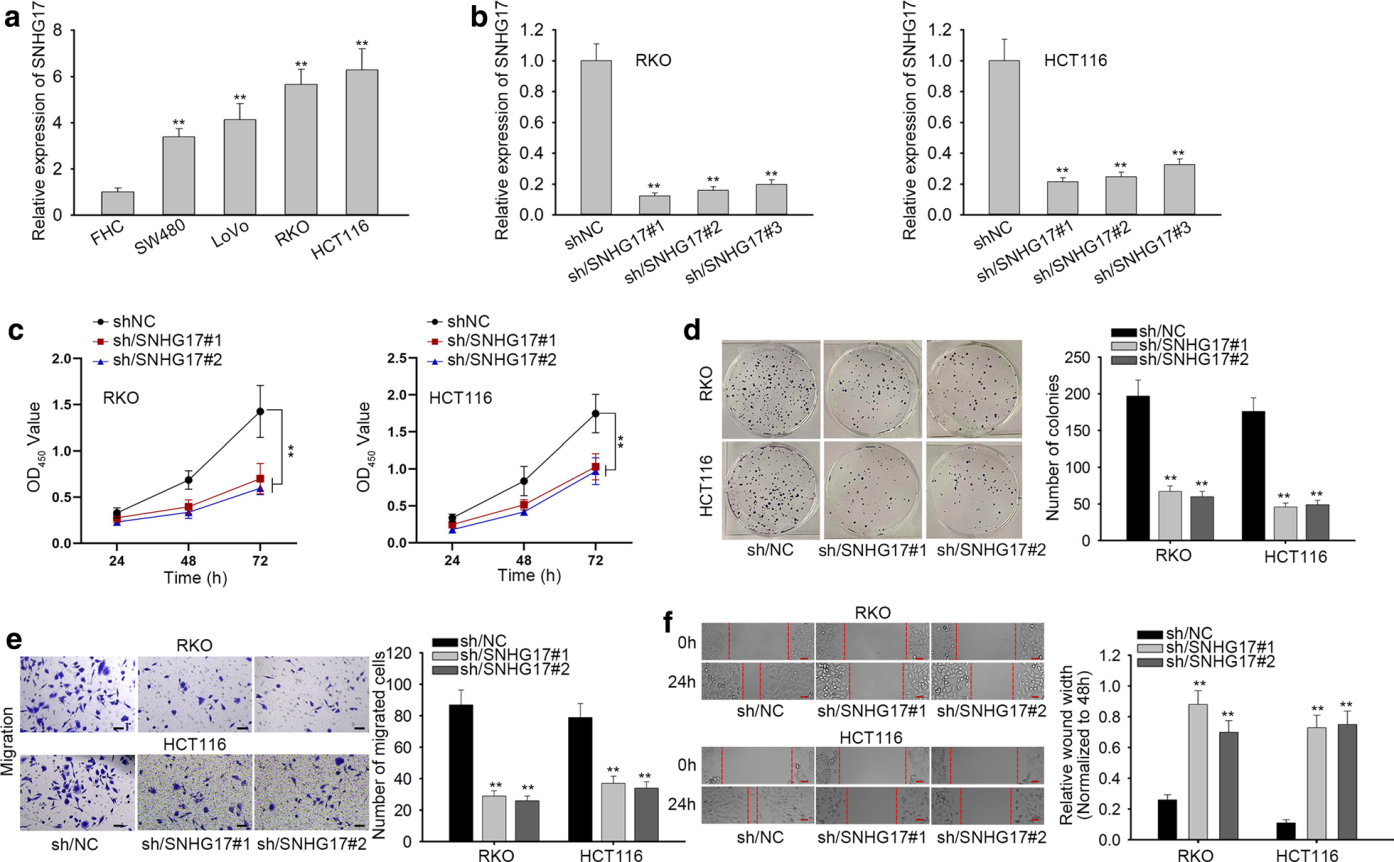
SNHG17 strengthens the viability, proliferation and migration of CRA cells. **a** The expression of SNHG17 was examined by RT-qPCR in CRA cell lines (SW480, LoVo, RKO and HCT116) and human colon epithelial cell line FHC. **b** The interference efficiency of sh/SNHG17#1&#2&#3 was tested in RKO and HCT116 cells. **c**, **d** CCK-8 assay and colony formation assay were carried out to examine cell viability and proliferation in cells with SNHG17 depletion. **e** Cell migration was evaluated by transwell assay after shRNA transfection. Scale bar, 100 μm. **f** The migratory ability of RKO and HCT116 cells was tested by wound healing assay. Scale bar, 100 μm. ^**^P < 0.01

To explore the role of SNHG17 in CRA, we used RT-qPCR to primarily examine SNHG17 expression in CRA cell lines (SW480, LoVo, RKO and HCT116) with human colon epithelial cell line FHC as control. The results revealed that SNHG17 was obviously overexpressed in CRA cells compared to FHC cell (Fig. 1a). Next, RT-qPCR analysis showed that SNHG17 was effectively down-regulated in RKO and HCT116 cells transfected with sh/SNHG17#1, sh/SNHG17#2 and sh/SNHG17#3 compared with shNC group (Fig. 1b). Furthermore, loss of-functional experiments were adopted to observe the effect of SNHG17 silencing on the biological behaviors of CRA cells. Through CCK-8 assay, we knew that the viability of CRA cells was greatly suppressed due to SNHG17 knockdown (Fig. 1c). Similarly, SNHG17 knockdown negatively regulated colony formation rate of CRA cells, which was clearly assessed by colony formation assays (Fig. 1d). Moreover, cell migration was examined by transwell and wound healing assays. As shown in Fig. 1e, the migratory capacity of two CRA cells was significantly restrained by silenced SNHG17. Meanwhile, SNHG17 knockdown also caused the broadening wound width (Fig. 1f). Based on above results, we concluded that silencing of SNHG17 represses cell viability, proliferation and migration in CRA.

### SNHG17 can interact with miR-23a-3p in CRA cells

**Fig. 2:**
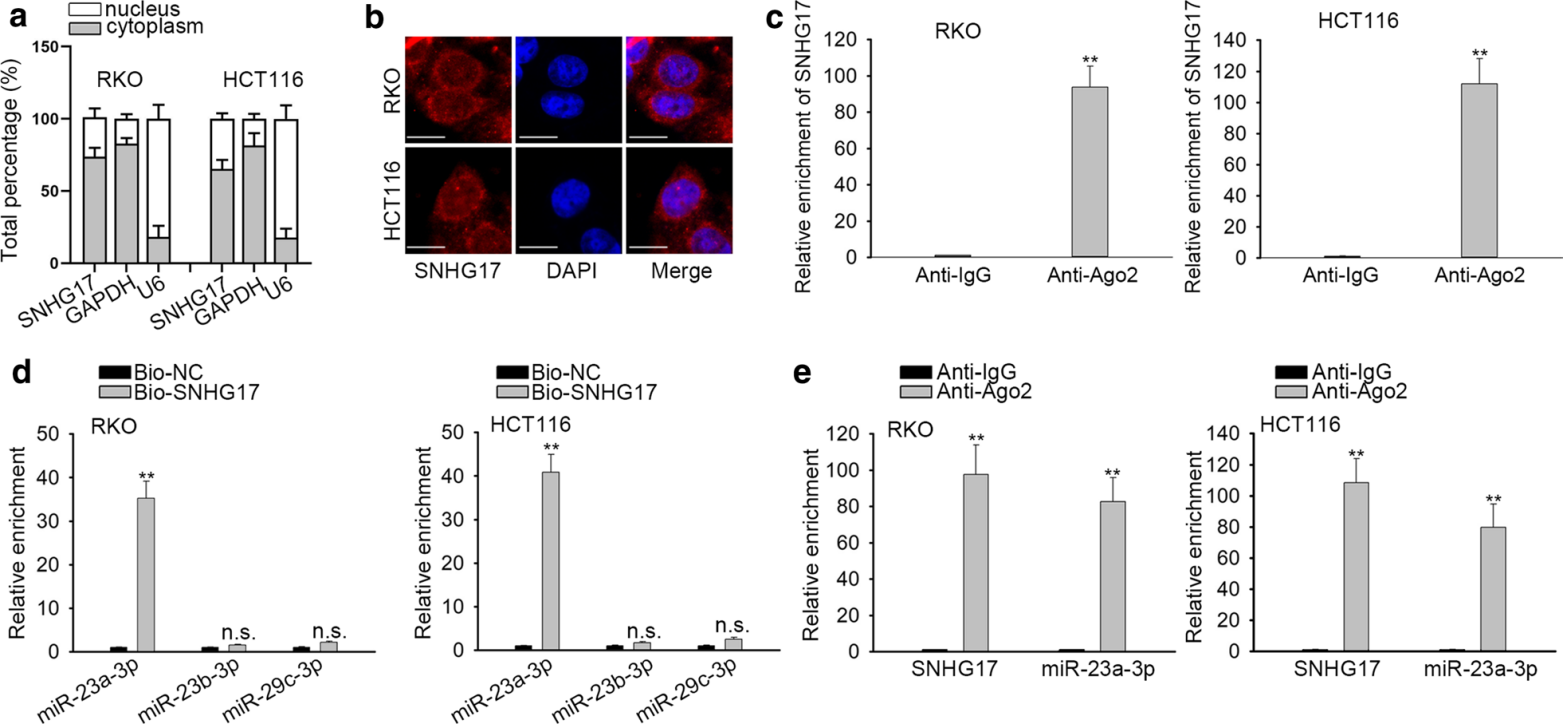
SNHG17 can interact with miR-23a-3p in CRA cells. **a**, **b** Subcellular fractionation assay and FISH assay (Scale bar, 20 μm) measured the localization of SNHG17 in CRA cells. **c** Ago2-RIP assay was used to assess the enrichment of SNHG17 in Anti-Ago2 group and Anti-IgG group. **d** The connection between SNHG17 and candidate genes was verified by RNA pull down assay. **e** Ago2-RIP assay was conducted in CRA cells with antibodies against Ago2 to confirm the interaction between SNHG17 and miR-23a-3p. ^**^P < 0.01. n.s.: no statistical significance

To identify the potential regulatory mechanism of SNHG17 in CRA cells, we firstly located SNHG17 in CRA cells through subcellular fractionation and FISH assay. According to the results, we determined that SNHG17 was mostly located in the cytoplasm of CRA cells (Fig. 2a, b). Cytoplasmic lncRNAs can act as competing endogenous RNAs (ceRNAs) in human cancers by sponging miRNAs to upregulate downstream mRNAs. However, whether SNHG17 plays the similar role in CRA cells has not been reported yet. Herein, we hypothesized that SNHG17 could function as a ceRNA in CRA. Next, Ago2-RIP assay was performed in CRA cells. The results disclosed that SNHG17 was enriched in Anti-Ago2 compared with that of Anti- IgG (Fig. 2c). Afterwards, we screened out underlying three miRNAs (miR-23a-3p, miR-23b-3p and miR-29c-3p) which possibly bound with SNHG17 from ENCORI (http://starbase.sysu.edu.cn/). RNA pull down assay was subsequently carried out to screen the candidate miRNA. As presented in Fig. 2d, miR-23a-3p enrichment was overtly high in Bio-SNHG17 group, while remaining two miRNAs had no significant enrichment, reflecting that SNHG17 could interplay with miR-23a-3p. To verify correlation of SNHG17 and miR-23a-3p, we performed Ago2-RIP assay and identified that SNHG17 and miR-23a-3p were both abundant in Anti-Ago2 complex (Fig. 2e). Finally, we found that miR-23a-3p expression was not significantly changed in response to SNHG17 downregulation (Additional file 2: Figure S1A). In conclusion, SNHG17 acts as a sponge of miR-23a-3p in CRA cells.

### SNHG17 promotes CRA cellular processes by inhibiting miR-23a-3p

**Fig. 3:**
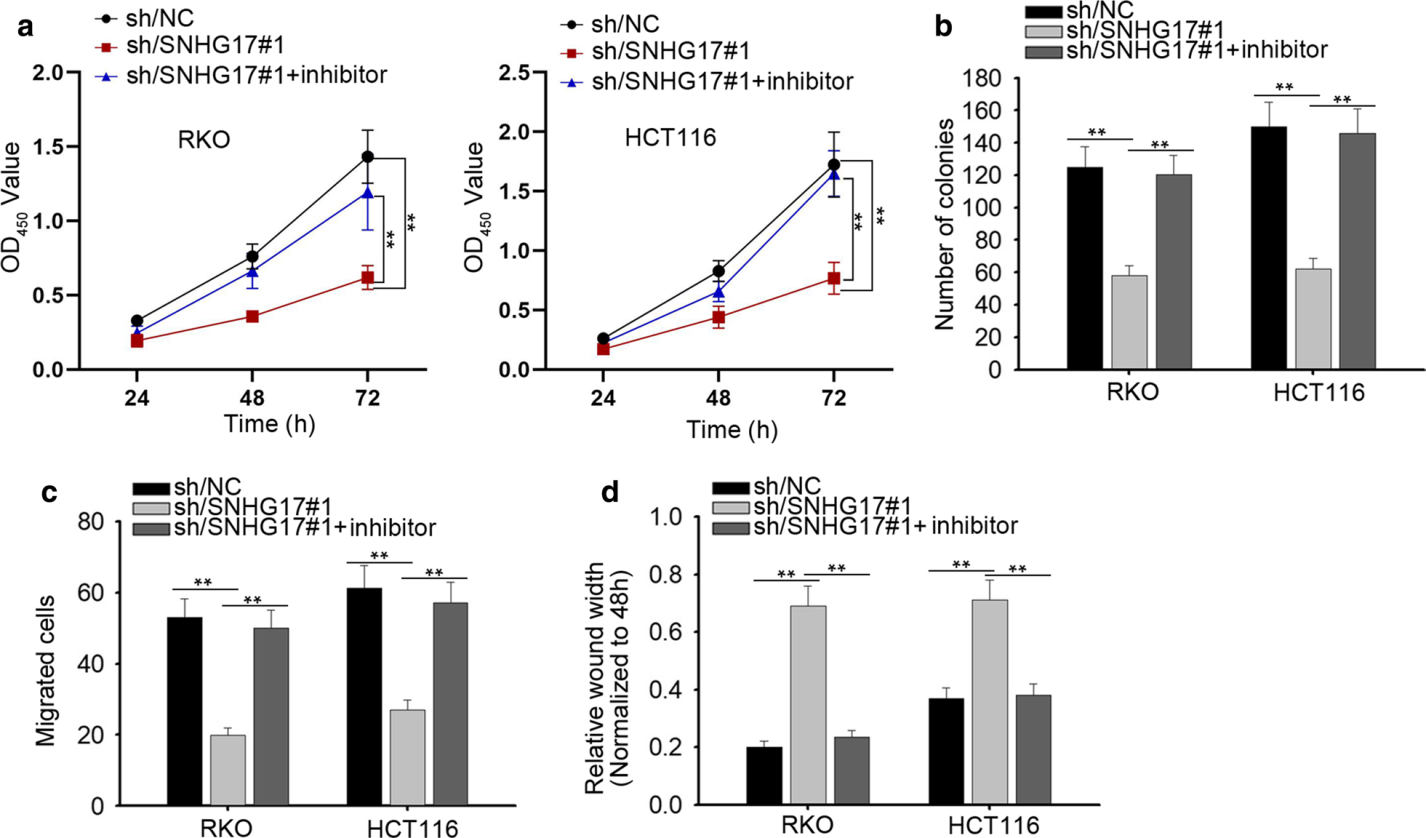
SNHG17 promotes CRA cellular processes by inhibiting miR-23a-3p. **a** CCK-8 assay tested the viability of CRA cells transfected with sh/SNHG17#1 and miR-23a-3p inhibitor. **b** The colony formation assay examined the CRA cell proliferation under indicated transfections. **c** Migratory abilities were assessed by transwell assay. **d** Wound healing assay was performed to evaluate the migratory capacity of two CRA cells. ^**^P < 0.01

To determine the impact of SNHG17/miR-23a-3p axis on the functions of CRA cells, we designed rescue assays. First, CCK-8 assay and colony formation assay were used to examine the viability and proliferation of CRA cells. As disclosed in Fig. 3a, b, the suppression on viability and proliferation caused by sh-SNHG17 was strengthened by miR-23a-3p inhibitor. Similarly, migration of CRA cells was initially hampered by SNHG17 silencing, but was elevated by miR-23a-3p knockdown (Fig. 3c). Wound healing assay also exhibited the same rescue effect. The wound width was increased with the down-regulation of SNHG17, later was narrowed with the silencing of miR-23a-3p (Fig. 3d). Taken together, silencing miR-23a-3p abolished the inhibitory effect of SNHG17 knockdown on the biological behaviors of CRA cells, indicating that SNHG17 promotes CRA progression by inhibiting miR-23a-3p.

### SNHG17 regulates CXCL12 through competitively binding with miR-23a-3p

**Fig. 4:**
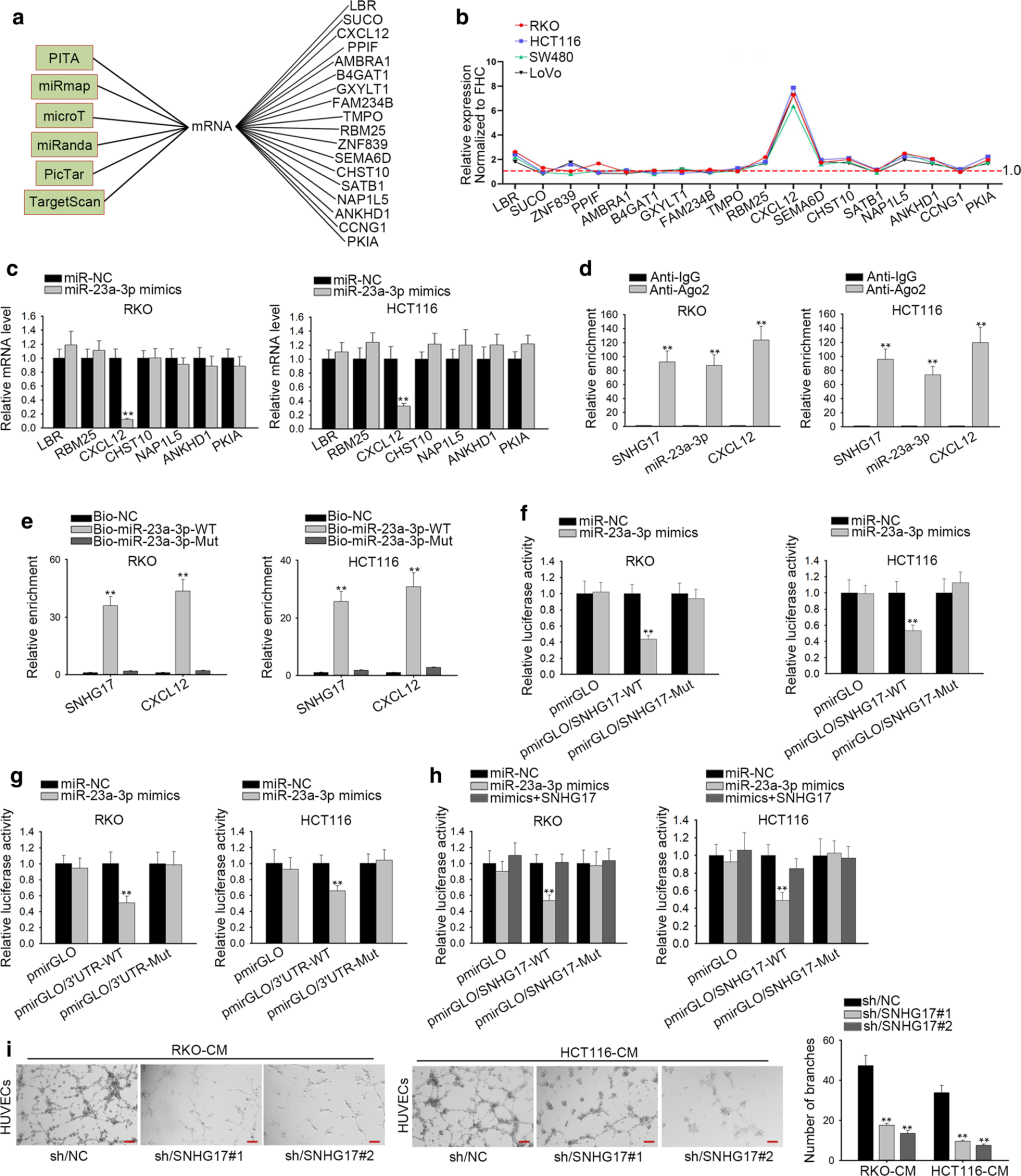
SNHG17 upregulates CXCL12 through absorbing miR-23a-3p. **a** The PITA, miRmap, microT, miRanda, PicTar and TargetScan databases were used to predict underlying 18 mRNAs genes. **b** RT-qPCR tested the expression of 18 mRNAs in CRA cell lines compared to normal cell line FHC. **c** The expression of seven candidate mRNAs was assessed by RT-qPCR in the case of miR-23a-3p mimics. **d**, **e** RIP assay and RNA pull down assay confirmed the association among SNHG17, miR-23a-3p, CXCL12. **f**, **g**. Luciferase reporter assays were used to confirm the interaction of miR-23a-3p with SNHG17 or CXCL12. **h** The luciferase activity of CXCL12-WT or CXCL12-Mut was measured in cells co-transfected with miR-23a-3p mimics and pcDNA3.1/SNHG17 by luciferase reporter assay.** i** Tube formation assay verified the effect of silenced SNHG17 on the tube formation of CRA cells. Scale bar, 100 μm. **P < 0.01

To explore the downstream genes of miR-23a-3p, we used databases like PTTA, miRmap, microT, miRanda, PicTar and TargetScan to screen out 18 candidate mRNAs which could bind with miR-23a-3p (Fig. 4a). On the basic of this data, RT-qPCR assessed the expression of 18 candidate mRNAs in CRA cell lines compared to FHC cell line. As shown in Fig. 4b, LBR, RBM25, CXCL12, CHST10 and NAP1L5, ANKHD1 and PKIA presented strong expression in CRA cells compared with FHC cells. Afterwards, we observed the alteration on the expression of above seven mRNAs in cells transfected with miR-23a-3p mimics. The results revealed CXCL12 was distinctly down-regulated by contrast with miR-NC group, while other six mRNAs had no significant alteration (Fig. 4c). Meanwhile, silencing of SNHG17 also led to the significant downregulation of CXCL12 (Additional file 2: Figure S1B). To further obtain the evidence, we conducted RIP assay and RNA pull down assay. As shown in Fig. 4d, the results suggested that the SNHG17/miR-23a-3p/CXCL12 were co-existed in RNA induce-silencing complex (RISC). Likewise, the result of RNA pull-down reflected that SNHG17 and CXCL12 could be pulled down by bio-miR-23a-3p-WT compared to Bio-NC group and Bio-miR-23a-3p-Mut (Fig. 4e). Furthermore, the luciferase activities of wild-type SNHG17 and CXCL12 were declined by miR-23a-3p mimics compared with miR-NC group, while that of mutant type was almost not changed, demonstrating that miR-23a-3p could bind to SNHG17 and CXCL12 (Fig. 4f, g). More importantly, the reduced luciferase activity CXCL12 caused miR-23a-3p overexpression was fully reversed by the upregulation of SNHG17 (Fig. 4h). These findings reflected that SNHG17 up-regulated CXCL12 through adsorbing miR-23a-3p. Besides, previous reports had declared CXCL12 was closely associated with angiogenesis. Thus, we used tube formation assay to analyze the effect of SNHG17 knockdown on angiogenesis. As suggested in Fig. 4i, the angiogenesis in HUVECs was effectively suppressed after SNHG17 was silenced. In a word, SNHG17 augments CXCL12 expression in CRA cells by sequestering miR-23a-3p.

### The effects of SNHG17/CXCL12 axis on the biological processes of CRA cells

**Fig. 5:**
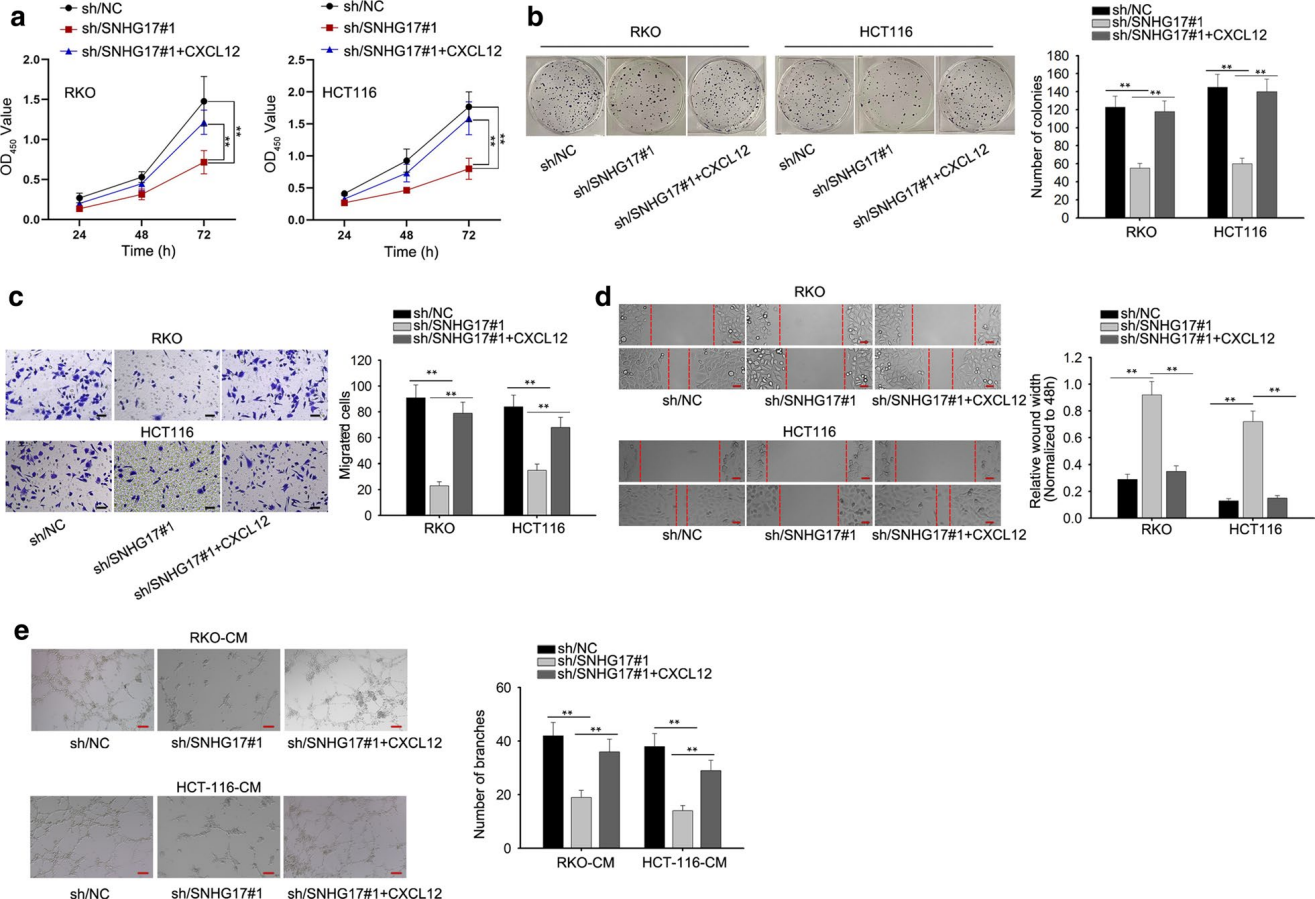
The effects of SNHG17/CXCL12 axis on the biological processes of CRA cells. **a**, **b**. The proliferation ability of transfected CRA cells were assessed by CCK-8 and colony formation assays. **c**, **d**. The migratory ability of CRA cells were detected via transwell assay and wound healing assay. Scale bar, 100 μm. F. Tube formation assay was performed to investigate the angiogenesis in CRA cells. Scale bar, 100 μm. ^**^P < 0.01

In order to learn the effects of SNHG17/miR-23a-3p/CXCL12 axis on biological behaviors of CRA cells, rescue assays were designed and performed in CRA cells. Firstly, CXCL12 was overexpressed in two CRA cells with pcDNA3.1/CXCL12 vector (Additional file 2: Figure S1C). In CCK-8 and colony formation assays, we observed that the reduced viability and proliferation of SNHG17-down-regulated CRA cells were strengthened by CXCL12 (Fig. 5a, b). Likewise, the result of transwell assay indicated SNHG17 knockdown restrained cell migration, which was totally abolished by CXCL12 up-regulation (Fig. 5c). The result of wound healing assay was consistent with that of transwell assay. As illustrated in Fig. 5d, CXCL12 overexpression abolished the inhibitory effect of SNHG17 silencing on cell migration. Tube formation assays suggested that the suppressive effect of sh-SNHG17 on angiogenesis was abolished by CXCL12 (Fig. 5e). Collectively, SNHG17 promotes the proliferation and migration of CRA cells by modulating CXCL12-mediated angiogenesis.

## Discussion

Previous investigations have shown that SNHG17 could exert as a biological participant in different types of human cancers. For example, SNHG17 is abnormally expressed in gastric cancer cells and predicts prognosis [19]. Likely, Xu et al. also demonstrated that SNHG17 silence effectively retarded the progression of non-small-cell lung cancer (NSCLC) [20]. Moreover, SNHG17 is discovered to participate in the development of breast cancer to become a useful and valuable biomarker [17]. In this study, the high expression of SNHG17 was firstly determined in CRA cells. Meanwhile, we also explored the effect of SNHG17 knockdown on cell proliferation and migration. The results revealed that SNHG17 depletion inhibited cell viability, proliferation and migration. A previous study has elucidated that SNHG17 can promote cell proliferation in colorectal cancer by downregulating p57 [21]. Similarly, we also uncovered that SNHG17 promoted CRA cell proliferation in the current study. Thus, we summarized that SNHG17 potentially exerts oncogenic functions in CRA.

Given that lncRNAs can regulate gene expression and exert functions through ceRNA networks. In a ceRNA network, lncRNAs can act as sponges for miRNAs and thus release the downstream mRNAs. SNHG17 has also been demonstrated to be a ceRNA in several cancer types. However, our current study is the first one to unveil the ceRNA role of SNHG17 in CRA. MiRNAs are usually deemed to modulate tumorigenesis by interplay with lncRNAs or mRNAs [22–24]. In this study, we uncovered that miR-23a-3p could interact with SNHG17 and form a ceRNA network in CAR cells. In regard to miR-23a-3p, Zhao et al. [25] express that downregulation of miR-23a-3p activated apoptosis and affected the cell cycle in acute myeloid leukemia. Besides, miR-23a-3p regulated lncRNA NEAT1 is confirmed to effect melanoma cancer progression [26]. In this study, we could discover miR-23a-3p played a tumor-inhibiting role in CRA. From the analysis of mechanism, SNHG17 contributed to promoting CRA progress by inhibiting miR-23a-3p. Here, we observed that the inhibition of CRA cell proliferation and migration induced by SNHG17 silencing could be reversed by miR-23a-3p inhibitor. Hereto, we determined that SNHG17 promotes CRA progression by inhibiting miR-23a-3p.

Evident reporters have showed that tumor growth has close correlation with angiogenesis [27, 28]. In this study, we also found that suppressive effect of SNHG17 knockdown on the angiogenesis of CRA-induced HUVECs. Combining with the mechanism investigation, we confirmed that CXCL12 was the downstream target of miR-23a-3p. Importantly, CXCL12 has been reported to affect the angiogenesis due to its angiogenic properties. For instance, CXCL12 actively regulates angiogenesis by stimulating tube formation [29, 30]. CXCL12/CXCR4 signal pathway participates in tumor progression and metastasis and survival, except for angiogenesis [31]. Similarly, the current experiences also revealed that CXCL12 abolished the suppressive effects of SNHG17 knockdown on angiogenesis in CRA cells. Meanwhile, CXCL12 also reversed the inhibition influence on cell proliferation and migration cause by silencing SNHG17. Therefore, we concluded that SNHG17 accelerates cell proliferation and migration in CRA by sponging miR-23a-3p to modulate CXCL12-mediated angiogenesis.

## Conclusion

In conclusion, this study explored a novel pathway of SNHG17/miR-23a-3p/CXCL12 in CRA. In detail, SNHG17 facilitates the progression of CRA by miR-23a-3p/CXCL12 axis. The findings suggest that SNHG17 may be a novel and promising target for CRA treatment.

## Supplementary information

**Additional file 1: Table S1.** Sequences for PCR primers and transfection plasmids.

**Additional file 2: Figure S1.** A. The expression of miR-23a-3p was measured in CRA cells with SNHG17 knockdown. B. CXCL2 mRNA level in cells transfected with SNHG17-specific shRNAs. C. CXCL12 overexpression induced by pcDNA3.1/CXCL12 vector was confirmed by RT-qPCR. ^**^P < 0.01.

## Data Availability

Not applicable.

## Abbreviations

lncRNAs: Long non-coding RNAs
mRNA: Messenger RNA
ceRNAs: Competing endogenous RNAs
miRNA: MicroRNA
CRA: Colorectal adenocarcinoma
SNHG17: Small nucleolar RNA host gene 17
CXCL12: C-X-C motif chemokine ligand 12
Wt: Wild-type
Mut: Mutant
RT-qPCR: Quantitative real-time PCR
RIP: RNA immunoprecipitation
SD: Standard deviation
