# Supplementary figures and images for: SNHG17 promotes the proliferation and migration of colorectal adenocarcinoma cells by modulating CXCL12-mediated angiogenesis
Source: Cancer Cell Int. 2020 Nov 26;20:566. doi: 10.1186/s12935-020-01621-0 (PMC7690009; doi:10.1186/s12935-020-01621-0)

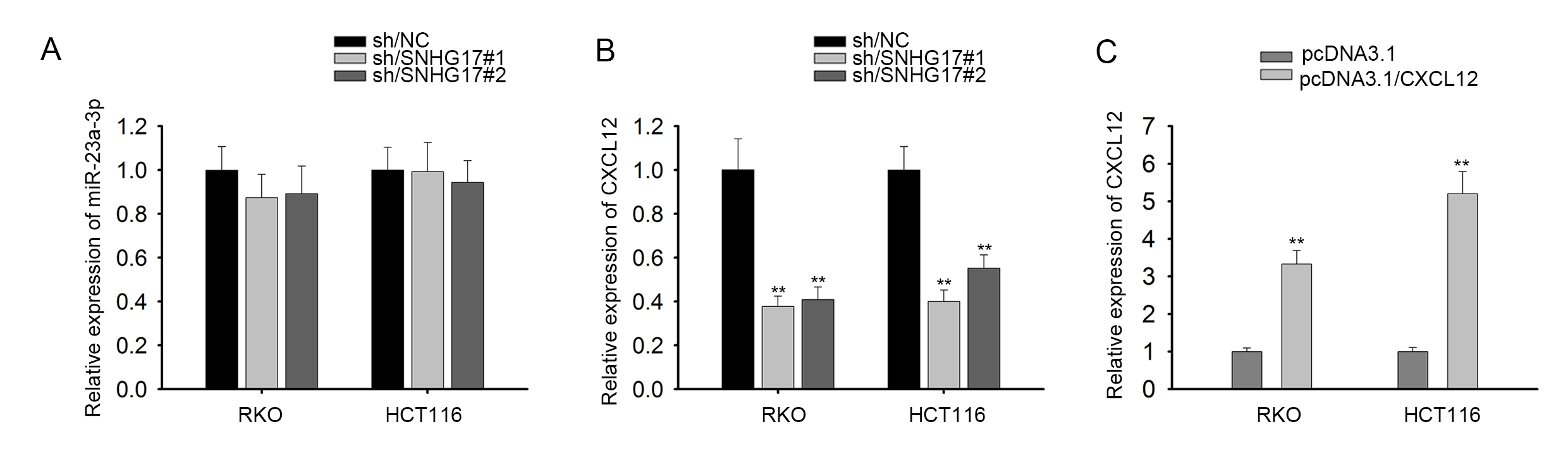

Supplement: Supplementary file 2 — Additional file 2: Figure S1. A. The expression of miR-23a-3p was measured in CRA cells with SNHG17 knockdown. B. CXCL2 mRNA level in cells transfected with SNHG17-specific shRNAs. C. CXCL12 overexpression induced by pcDNA3.1/CXCL12 vector was confirmed by RT-qPCR. **P < 0.01. [file 12935_2020_1621_MOESM2_ESM.tif]
